# Net ownership, utilization and malaria burden in the context of Piperonyl butoxide-LLINs intervention in western Kenya

**DOI:** 10.1371/journal.pone.0329114

**Published:** 2025-08-18

**Authors:** Job Oyweri, Patrick O. Onyango, Maxwell G. Machani, Josephat Bungei, Sammy Cheruiyot, Yaw A. Afrane, Ming-Chieh Lee, Daibin Zhong, Guofa Zhou, John Githure, Harrysone Atieli, Guiyun Yan

**Affiliations:** 1 Department of Zoology, Maseno University, Kisumu, Kenya; 2 International Centre of Excellence for Malaria Research, Tom Mboya University, Homabay, Kenya; 3 Centre for Global Health Research, Kenya Medical Research Institute, Kisumu, Kenya; 4 Faculty of Science and Technology, University of Nairobi, Nairobi, Kenya; 5 Department of Medical Microbiology, University of Ghana Medical School, Accra, Ghana; 6 Department of Population Health and Diseases Prevention, University of California, Irvine, California, United States of America; Freelance Consultant, Myanmar, MYANMAR

## Abstract

**Background:**

Pyrethroid-treated nets have reduced malaria in endemic areas, but insecticide resistance has hindered progress, prompting WHO to recommend piperonyl butoxide (PBO) based long-lasting insecticidal nets (LLINs). Kenya adopted PBO nets, but their usage is not well documented. This study aims to assess the uptake and effect of PBO nets versus pyrethroid-only LLINs (pyrethroid-LLINs) on malaria transmission in Western Kenya.

**Methods:**

A cross-sectional study was conducted in Muhoroni Sub-County, Kisumu County, one year after net distribution between November and December 2023. Twelve villages were randomly selected, comprising 380 households and divided into two intervention arms of six villages. Group 1, with 181 households, received pyrethroid-LLIN, while Group 2, with 199 households, received PBO-LLINs. Data on net ownership and usage was collected using a standardized semi-structured questionnaire. Finger prick blood smears were collected on slides for microscopic examination, while dry blood spots (DBS) on filter paper were collected for real-time PCR (RT-PCR) diagnosis of *Plasmodium* infection in both intervention groups. Logistic regression was used to identify factors influencing net ownership and malaria prevalence, while a generalized linear model assessed factors affecting net usage. The χ² test was used to evaluate differences in demographic characteristics between the intervention arms.

**Results:**

Overall, higher net ownership and usage was recorded in PBO-LLINs group compared to the pyrethroid-LLIN group after one year of net distribution. Among households, 89% in the pyrethroid-LLIN group and 96% in the PBO-LLIN group owned at least one net. Net usage was 80.2% in the pyrethroid-LLIN group and 87.6% in the PBO-LLIN group. Net attrition rate was 17.9% in pyrethroid-LLIN and 7.6% for PBO-LLIN group. Households with nets were 1.3 times more likely to use them [aOR=1.338 (95% CI = 1.224–1.462), p < 0.001). Bed ownership was 50.8% in the pyrethroid-LLIN group and 55.3% in the PBO-LLIN group. Not owning a bed decreased the likelihood of net usage by 13.3% [aOR=0.867 (95% CI = 0.816–0.920), p < 0.001]. Between the two groups, 75.7% of households in the pyrethroid-LLIN group and 66.8% in the PBO-LLIN group reported bedbug infestations in their homes. Bedbug infestation significantly affected net ownership (p = 0.018). Malaria parasite prevalence was 34.7% by qPCR in the pyrethroid-LLIN group and 29.0% in the PBO-LLIN group, with a prevalence ratio of 0.84 (95% CI: 0.65–1.03).

**Conclusion:**

The study observed higher bednet ownership, usage, and lower malaria prevalence in the PBO-LLIN clusters compared to the pyrethroid-LLIN clusters. However, bedbug infestations and insufficient sleeping beds hindered net ownership and usage, limiting their overall effectiveness. These findings highlight the need for National Malaria Control Programs (NMCPs) to enhance community education on proper net use and maintenance through targeted initiatives, such as household visits and local workshops. Furthermore, incorporating bedbug control strategies and regular surveillance will improve compliance and net usage. Addressing these challenges will maximize the impact of PBO-LLINs and other next-generation nets, strengthening malaria control and elimination efforts in both urban and rural settings.

## Introduction

The core vector control measures, universal LLIN coverage, targeted indoor residual spraying (IRS), and prompt diagnosis and treatment, have been scaled up in western Kenya since the early 2000s. These control measures have been reported to effectively reduce malaria burden, but not sufficient to eliminate malaria. According to WHO (2022) [1], Kenya recorded approximately 12,011 malaria-related deaths from 3,419,698 reported cases. The 2021 Kenya Malaria Indicator Survey reported a national malaria prevalence of 5.6%, with the highest burden in the lake-endemic region 18.9%. Among children under five, malaria prevalence was 3%, with rural areas being more affected than urban ones, and the highest prevalence (19%) was observed among children aged 6 months to 14 years in the lake-endemic region [2]. Pregnant women in this region experience malaria prevalence ranging from 9% to 18% [3]. A key challenge contributing to persistent malaria transmission is insecticide resistance in local vector populations [4–6]. Growing concerns that resistance may be undermining vector control efforts and hindering malaria reduction have highlighted the urgent need for more effective insecticide resistance management strategies.

Vector control methods including larval source management (LSM), IRS and LLINs have averted at least 2.1 billion cases and 11.7 million deaths globally since 2,000 [7,8]. Through LSM, larvae are killed via habitat modification, manipulation, chemical larviciding and biological control. Its success relies on concerted community participation of identifying larval breeding habitats [9]. Indoor residual spraying method applies insecticides to interior walls where mosquitoes rest, effectively reducing malaria transmission by targeting indoor-resting vectors. In Western Kenya, pyrethroids were exclusively used for IRS with one round per year until 2012. As from 2013, the WHO recommended the use of carbamates and organophosphates for IRS to manage pyrethroid resistance [10]. In 2019, a newly prequalified insecticide, Fludora fusion (neonicotinoid + pyrethroids) was piloted in Homabay, Western Kenya. In 2021, the insecticide was further sprayed in eight sub counties of Homabay and six in Migori, Western Kenya [11]. Long-lasting insecticide-treated nets reduce human-vector contact by creating a physical barrier, inhibiting blood feeding potential, repel or kill mosquitoes [12]. However, pyrethroid resistance has persisted due to continuous use of pyrethroids on LLINs. Incorporating PBO into LLINs is a promising strategy to mitigate pyrethroid resistance and enhance vector control, particularly in areas with high levels of resistance [8,13,14].

In Kenya, the National malaria control program (NMCP) has adopted these PBO-treated nets in high-transmission areas with significant insecticide resistance to supplement existing pyrethroid-LLINs [15]. However, the efficacy of these novel nets in preventing malaria in Kenya is yet to be fully determined. Moreover, LLIN usage at national level remains below universal coverage, partly due to low ownership [8]. The gap between LLIN access and usage highlights the critical role of human behavior in malaria transmission, treatment, and control.

Community-wide protection depends on collective ownership and utilization, with ≥80% usage needed to protect the entire population, including non-users [2,12]. Statistically, 70% of households in sub-Saharan Africa possessed at least one insecticide-treated net (ITN) by 2022, a significant increase from about 5% in 2000. The proportion of the population sleeping under an ITN rose from roughly 2% to 49%. However, since 2015, there has been no significant increase in overall access to ITNs and usage [8]. According to the Kenya Demographic and Health Survey (2022), national net ownership was 54.2%, with universal coverage of 37.1%. In Kisumu, Western Kenya, net ownership was 76% with 47% universal coverage. Nationally, net usage was recorded at 42.7% with 77% usage among children under 5 years old and 75% usage among pregnant women. The lake-endemic zone recorded net usage rates of 80.1% among children under five and 82.2% among women [16]. Despite the widespread distribution of ITNs in malaria-endemic regions, significant gaps remain in understanding their actual usage and retention at the household level. Several factors have been reported to influence net usage, including outdoor night-time activities, cultural beliefs, lack of proper beds, net quality perceptions, and preferences for specific net types [2,17–21]. Examining net ownership, usage patterns, and the barriers affecting them is crucial for assessing the effect of LLINs, particularly the newly introduced PBO nets, which are yet to be fully implemented.

## Materials and methods

### Study site and population

The study was carried in Muhoroni sub-county in Kisumu County, western Kenya, an area that covers 425.3 km^2^. According to the 2019 Kenya National Bureau of Statistics, Muhoroni sub-county has a population of 154,116 residents [22]. The region experiences hot and wet climate averaging approximately 20^o^C and rainfall of 1500 mm. The local residents are farmers who predominantly depend on sugarcane and rice farming for subsistence [23–25]. Malaria transmission in the study area has been described by Zhou et al. [26]. The survey was conducted across 12 villages (clusters), six in each of the two intervention arms of PBO-LLINs and standard pyrethroid-LLINs.

Using Cochran’s formula [27], sample size was determined as follows:


$$S=\frac{Z^{2}P(1-P)}{d^{2}}\to \frac{1.96^{2}\times 0.47\times 0.53}{0.05^{2}}=382.78\approx 383$$

Where; S = Infinite sample size, Z = Z-score at 95% confidence level (1.96), P = Proportion of net coverage in Kisumu according to the Kenya Demographic and Health Survey in 2022, (0.47) (16), d = Precision 0.05.

Correction factor formula was applied as follows:


$$Samplesize(n)=\frac{S}{1+\left(\frac{s-1}{N}\right)}=\frac{383}{1+\left(\frac{383-1}{1200}\right)}=290.51\approx 291.$$

Where; N Represents the number of households in 12 villages (6 in each LLIN group). A village had 100 households. A 10% margin was added to account for potential household refusals and absenteeism during surveys yielding 30. Total expected sample size was at least 321 (both PBO and pyrethroid-LLIN intervention arms). Thus, a sample size of ≥161 per intervention arm was necessary.

### Study design

A cross-sectional comparative study design was carried out to determine net ownership, usage and malaria prevalence one-year post-nets distribution. In November 2022, community health promoters (CHPs) and field assistants distributed LLINs while adhering to the WHO universal coverage guidelines (1 LLIN per 2 people). The distributed nets were uniquely marked with a study abbreviation code (ICEMR) for proper identification. The CHPs conducted a one-week mobilization exercise to encourage residents to participate in the net distribution exercise. One week after net distribution, a mop-up exercise was done to issue nets to households that may have been left out due to absence. The distributed nets were PermaNet 2.0 (pyrethroid-LLIN) and PermaNet 3.0 (PBO-LLIN), both manufactured by Vestergaard, Switzerland. The residents were taken through proper LLIN hang and use practices before the beginning of the study and during distribution. A total of 380 randomly selected households consisting of 199 in the PBO-LLIN and 181 in the pyrethroid-LLIN arms were used. Global Positioning System coordinates were taken and demographic characteristics of the residents determined.

### Net ownership and usage survey

Between 15^th^ November and 5^th^ December 2023, surveys were conducted in the 380 households across the two intervention arms using a semi-structured questionnaire to assess net ownership and usage. Net ownership was measured as the percentage of households with at least one net at time of visit, while net usage was the proportion of people using a net the previous night [28,29]. Households were defined as units sharing a cooking place, eating table, sleeping space, and/or living under the same roof [22,30]. A household head was defined as a responsible adult member who could manage and make decisions related to the house. Interviews were conducted with the household head or a responsible adult if the head was unavailable. Key informants provided data on age, occupation, household members, malaria episodes in the past month, bed and/or sleeping spaces, net availability, previous night net usage, and net fabric material preferences. Interviewers verified the type and number of nets in each household. Interviews were also conducted to determine night-time outdoor activities (6 pm-4 am), the presence of bedbugs, their effect on net usage (i.e., require more nets, use less nets, or use same number of nets or not sure), and any bedbug control measures used.

### Parasitological data collection and processing

During household interviews, technicians collected finger-prick blood samples from willing study participants. Thick and thin blood smears were made on glass slides. Drops of blood were also collected on Whatman filter papers in form of dried blood spots (DBS). A total of 963 samples comprising of 450 in Pyrethroid-LLIN group and 513 in the PBO-LLIN group were collected. The samples were then transported to the International Centre of Excellence for Malaria Research laboratory at Tom Mboya University, Homabay, for parasite examination by microscopy and qPCR of the DBS as described by Ochwedo et al. [31].

Dried blood spots were punched aseptically and genomic DNA extracted using the Chelex method with minor modifications [32]. The RT-PCR was used to determine *plasmodium* infections on the 963 DBS samples respectively. The Primers and probes specific to *Plasmodium* species targeting 18S rRNA were used. A final reaction volume of 12 µL comprising of 2 µL parasite DNA, 6 µL of PerfeCTa® qPCR ToughMix™, Low ROX™ Master Mix (2X), 0.5 µL of species-specific probe, 0.4 µL of forward and reverse species-specific primers (10 µM) (Pf, Pm and Po), and 0.1 µL of double-distilled water. The thermal programme used was an initial step at 50 °C for 2 minutes, followed by 45 cycles of 95 °C for 2 minutes, 95 °C for 3 seconds, and 58 °C for 30 seconds.

### Ethics approval and consent to participate

Research approval was obtained from Maseno University Scientific and Ethics Review Committee (MUSEREC) under study number MUERC/00778/19 and the University of California, Irvine, Institutional Review Board (UCI IRB) and received authorization from the Ministry of Health, Kisumu, Kenya. From November 1^st^ to 12^th^ November 2022 written informed consent was sought from willing adults. Young children were also assented for by their parents/guardians. Regardless of their demographics, each resident had a chance to participate and drop out of the study at any time. The willing participant and a witness provided their name, signature and date. Guidelines for consenting and withdrawal were strictly followed.

### Inclusivity in global research

Additional information regarding the ethical, cultural, and scientific considerations specific to inclusivity in global research is included in the Supporting Information (SX Checklist).

### Data management and analysis

All collected data were cleaned, coded, and securely stored in Microsoft Excel. The data were then imported into SPSS software (Version 27) for analysis. Data normality was assessed using the Kolmogorov-Smirnov test. Logistic regression analysis was performed to identify factors influencing net ownership, as well as to assess the effect of net ownership and usage on malaria prevalence. A generalized linear model was used to determine factors affecting net usage. Differences in age between intervention groups were analyzed using the Mann-Whitney U test. The chi-square (χ²) test was applied to compare differences in gender distribution, household size, and household occupation across interventions. Statistical significance for all tests was set at p ≤ 0.05.

## Results

### Demographic characteristics of study population

Out of 181 household heads interviewed in pyrethroid-only LLINs group 59.7% (108/181) were female, while 40.3% (73/181) were male. In the PBO-LLIN group, 54.8% (109/199) were female and 45.2% (90/199) were male. Overall, there were more female household heads across both intervention groups, but the difference was not statistically significantly (χ² = 0.927, df = 1, p = 0.336). The mean age of household heads was 47.24 ± 18.05 (SD) years in the pyrethroid-only LLIN group and 44.17 ± 17.57 (SD) years in the PBO-LLIN group. The average family size was comparable between the two intervention arms (3.8 and 3.7 respectively). The majority of study participants were aged ≥15 years. The primary socioeconomic activity was farming/agricultural work, with 51.4% (93/181) of participants in the pyrethroid-LLIN group and 46.2% (92/199) in the PBO-LLIN group (Table 1).

**Table 1:** Summary of population age distribution and occupation.

Variable	Category	Intervention arms		*p*-value
		Pyrethroid-LLINs (n = 181 households)	PBO-LLINs (n = 199 households)	
Population age group	0-4 years	40/681 (5.87)	59/739 (7.98)	0.202
	5-14 years	182/681 (26.73)	208/739 (28.14)	
	≥15 years	459/681 (67.40)	472/739 (63.87)	
Household head occupation	Farmer/Agricultural worker	93 (51.4)	92 (46.2)	0.041
	Fishing	1 (0.6)		
	Office worker	7 (3.9)	7 (3.5)	
	Student	5 (2.8)	16 (8)	
	Security	2 (1.1)	2 (1)	
	Mining, factory, construction	1(0.6)	8 (4)	
	Unemployed	32 (17.7)	22 (11.1)	
	Trader/ business person	39 (21.5)	52 (26.1)	
	Others	1 (0.6)		

### LLINs ownership and usage

Overall LLIN ownership was higher for PBO-LLINs 96% (191/199) compared to pyrethroid-LLINs 89% (161/181). The average number of nets per household was higher in the PBO-LLIN group (1.8 vs. 1.6), resulting in better population net access (92.6% vs. 82.2%) (p = 0.524). A significant difference in net ownership was observed between the two groups after one year (χ² = 6.862, df = 1, p = 0.009). Of the PBO-LLINs and pyrethroid-LLINs distributed, 92.4% (342/370) and 82.1% (280/341) respectively were still in the households one-year post-distribution. Regarding fabric preference, 65.7% (119) of the pyrethroid-LLIN group preferred rough nets, while 34.3% (62) preferred soft nets. In the PBO-LLIN group, 11.6% (23) preferred rough nets and 88.4% (176) preferred soft nets.

Of the 181 households in the pyrethroid-LLIN group, 50.8% (92/181) owned a bed, compared to 55.3% (110/199) in the PBO-LLIN group. Among those without a bed, 84.3% (75/89) in the pyrethroid-LLIN group and 62.9% (56/89) in the PBO-LLIN group slept on mattresses on the floor; the rest used sofas or gunny bags. Net usage was higher in the PBO-LLIN group (87.6%) compared to the pyrethroid-LLIN group (80.2%) (p = 0.088); Table 2. Net usage was highest among individuals aged ≥15 years in both groups, with the lowest usage among children under 5 years in the pyrethroid-LLIN group and 5–14 years in PBO-LLIN group. Among households owning a bed, net usage was 90.4% in the PBO-LLIN group and 84.5% in the pyrethroid-LLIN group.

**Table 2:** Net usage across intervention arms.

Variable	Category		Pyrethroid-LLIN Frequency (%)	PBO-LLIN Frequency (%)	*p*-value
Net usage			483/602 (80.2)	621/709 (87.6)	0.088
Net usage across age group	0-4 years	Yes	23/37 (62.2)	47/55 (84.5)	<0.001
		No	14/37 (37.8)	8/55 (14.5)	
	5-14 years	Yes	117/157 (74.5)	160/200 (80)	
		No	40/157 (25.5)	40/200 (20)	
	≥15 years	Yes	343/408 (84.1)	414/454 (91.2)	
		No	65/408 (15.9)	40/454 (8.8)	

### Factors associated with net ownership and usage at household level

Bedbugs infestation affected bed net ownership and usage across both intervention arms. In the Pyrethroid-LLIN group, 75.7% (137/181) households, and in the PBO-LLIN group, 66.8% (133/199) households reported seeing bedbugs. Among them, 58.6% (106/181) in the Pyrethroid-LLIN group and 49.7% (99/199) in the PBO-LLIN group attempted bedbug control strategies. The following variables were included in a logistic regression model to predict net ownership (Table 3): bedbug infestation, efforts to control bedbugs, and intervention type. Households that reported seeing bedbugs were 75.3% less likely to own a net compared to those that had not seen bedbugs [aOR 0.247 (95% CI = 0.078–0.783), p = 0.018]. Those attempting to control bedbugs were 3.8 times more likely to own a net [aOR 3.764 (95% CI = 1.573–9.004), p = 0.003]. Other reasons for not owning a net in the pyrethroid-LLIN arm included: 20% gave nets to needy relatives, 45% had nets stolen, and 25% found nets uncomfortable. In the PBO-LLIN group, 37.5% repurposed nets, 25% had nets stolen, and 12.5% reported torn nets. Households in the PBO-LLIN intervention arm were 3.5 times more likely to own a net compared to the pyrethroid-LLIN arm [aOR 3.528 (95% CI = 1.265–9.840), p = 0.016].

**Table 3:** Factors affecting bednets ownership at household level.

Variable	Total variable (%)/ mean±SD n = 380	Coefficient	aOR (95% CI)	*p*-value
Household head age	45.6 ± 17.843	0.005	1.005 (0.982-1.029)	0.665
Sex of household head (male)	163 (42.9)	0.121	1.129 (0.477-2.672)	0.782
Household population	3.74 ± 1.792	−0.062	0.939 (0.748-1.179)	0.590
Owning a Bed (yes)	202 (53.2)	0.188	1.207 (0.517-2.818)	0.664
Outdoor activities (yes)	361 (95)	0.375	1.454 (0.273-7.735)	0.660
Seen Bed bugs (yes)	270 (71.1)	−1.399	0.247 (0.078-0.783)	0.018
Efforts to control bedbugs (yes)	205 (53.9)	1.325	3.764 (1.573-9.004)	0.003
Intervention used (Type of net) (PBO-LLIN)	199 (52.4)	1.261	3.528 (1.265-9.840)	0.016
Preferred net fabric texture (smooth)	238 (62.6)	−0.252	0.778 (0.300-2.015)	0.605
Constant		2.151	8.592	0.079

Dependent variable: Net ownership.

Model: (Intercept), household age, household head sex, population, bed ownership, engagement in outdoor activities, seen bedbugs, efforts to control bedbugs, intervention used (Type of net) and net fabric texture preferred.

A Generalized Linear Model (GLM) was used to predict net usage at the household level (Table 4). Households owning a net were 1.3 times more likely to use it [aOR=1.338 (95% CI = 1.224–1.462), p < 0.001]. Not owning a bed reduced the likelihood of net usage by 13.3% [aOR=0.867 (95% CI = 0.816–0.920), p < 0.001]. Households not making efforts to control bedbugs were more likely not to use their nets compared to those attempting control measures [aOR=1.075 (95% CI = 1.002–1.153), p = 0.045]. When asked about the potential impact of bedbug infestations on net usage, 105 (58%) and 101 (50.8%) households cited that they could use same number of nets in pyrethroid and PBO-LLINs groups respectively. Further, 16 (8.8%) and 26 (13.1%) reported they could use more nets while 26 (14.4%) and 46 (23.1%) could use less nets respectively. The rest of the households were unsure of how bedbugs could affect their net use-behaviour.

**Table 4:** Factors affecting LLINs usage at household level.

Variable	Details	Total variable (%) /mean±SD n = 341	Coefficient	aOR (95% CI)	*p*-value
Net ownership	Proportion of people having access to a net	1.06 ± 0.409	0.291	1.338 (1.224-1.462)	<0.001
Age	Household head age	45.6 ± 17.488	−0.001	0.999 (0.997-1)	0.147
Sex	Female	193 (56.6)	−0.015	0.985 (0.926-1.047)	0.631
	Male	148 (43.4)	0	1	
Occupation	Farmer/agricultural worker	169 (49.6)	−0.226	0.798 (0.472-1.346)	0.397
	Fishing	1 (0.3)	−0.073	0.930 (0.448-1.931)	0.845
	Office worker	12 (3.5)	−0.21	0.811 (0.469-1.401)	0.452
	Student	18 (5.3)	−0.198	0.820 (0.479-1.404)	0.47
	Security	4 (1.2)	−0.172	0.842 (0.472-1.502)	0.561
	Mining, factory, Construction	9 (2.6)	−0.138	0.871 (0.505-1.502)	0.619
	Unemployed	46 (13.5)	−0.205	0.815 (0.480-1.381)	0.447
	Trader/business person	81 (23.8)	−0.211	0.810 (0.479-1.370)	0.432
	Others	1 (0.3)	0	1	
Household population	Population	3.8 ± 1.765	0.005	1.005 (0.985-1.025)	0.6
Reported episodes of malaria within a month	No	148 (43.4)	0.06	1.062 (0.998-1.130)	0.056
	Yes	193 (56.6)	0	1	
Bed ownership	No	154 (45.2)	−0.143	0.867 (0.816-0.920)	<0.001
	Yes	187 (54.8)	0	1	
Engagement in outdoor activities	No	16 (4.7)	0.059	1.061 (0.925-1.215)	0.4
	Yes	325 (95.3)	0	1	
Seen bedbugs in households	No	105 (30.8)	−0.002	0.998 (0.923-1.079)	0.956
	Yes	236 (69.2)	0	1	
Effect of bedbug infestation on net usage	Same net usage	188 (55.1)	0.061	1.063 (0.964-1.171)	0.223
	More net usage	42 (12.3)	0.023	1.023 (0.899-1.164)	0.726
	less net usage	61 (17.9)	0.027	1.027 (0.914-1.156)	0.646
	Not sure	50 (14.7)	0	1	
Efforts to control bedbugs	No	153 (44.9)	0.072	1.075 (1.002-1.153)	0.045
	Yes	188 (55.1)	0	1	
Intervention	Pyrethroid-LLIN	154 (45.2)	−0.031	0.970 (0.900-1.044)	0.41
	PBO-LLIN	187 (54.8)	0	1	
Preferred fabric texture quality	Rough	124 (36.4)	−0.014	0.986 (0.914-1.065)	0.728
	Soft	217 (63.6)	0	1	

Determination by GLM.

Dependent variable: Net usage (Proportion of people sleeping under net) at household level.

Model: (Y intercept), net ownership, household head age, sex of the household head, occupation of the household head, reported episodes of malaria within a month, bed ownership, engagement in outdoor activities, seen bedbugs in households, effect of bedbug infestation on net usage, efforts to control bedbugs, net type and preferred fabric texture quality.

At the individual level, intervention type and age group significantly affected LLIN usage (Table 5).

**Table 5:** Factors affecting net usage at the individual level.

Variable	Details	Frequency (%) n = 1311	Coefficient	aOR (95% CI)	*p*-value
Intervention type	PBO-LLIN	709 (54.1)	0.804	2.235 (1.722-2.899)	<0.0001
	Pyrethroid-LLIN	602 (45.9)	0	1	
Sex	Male	621 (47.4)	0.050	1.051 (0.813-1.359)	0.703
	Female	690 (52.6)	0	1	
Age group					0.001
	5-14 years	359 (27.4)	0.065	1.068 (0.652-1.749)	0.795
	≥15 years	878 (67)	0.680	1.975 (1.232-3.166)	0.005

Determination by logistic regression, Dependent variable: Net usage, Independent Variables: Intervention type, Sex, Age group

### Malaria infection prevalence across age groups

Out of 450 samples in the pyrethroid-LLIN group, malaria parasite prevalence was 15.8% (71) by microscopy and 34.7% (156) by qPCR. In the PBO-LLIN group, out of 513 samples, prevalence was 10.9% (56) by microscopy and 29% (149) by qPCR. Submicroscopic infections were 18.9% (85/450) in the pyrethroid-only group and 18.1% (93/513) in the PBO-LLIN group. *Plasmodium falciparum* (Pf) accounted for over 95% of infections, with the remainder being mixed infections of Pf and *Plasmodium malariae* (Pm) or *Plasmodium ovale* (Po). A significant difference in malaria prevalence by microscopy was recorded across age groups (p < 0.001). Out of the total participants (n = 450) tested by microscopy in pyrethroid-only LLIN group, 27.3% (9/33) aged 0–4 years, 25.8% (33/128) aged 5–14 years, and 10% (29/289) aged 15 years and older tested positive for the parasites. In the PBO-LLIN group (n = 513), 8% (4/50) aged 0–4 years, 16.8% (28/167) aged 5–14 years, and 8.1% (24/296) aged 15 years and older tested positive by microscopy. Overall, no significant difference in confirmed malaria prevalence by qPCR was recorded (prevalence ratio of 0.84 (95% CI: 0.65–1.03; p = 0.129). Across age groups, in the Pyrethroid-LLIN group, 42.4% (14/33) aged 0–4 years, 37.5% (48/128) aged 5–14 years, and 32.5% (94/289) aged 15 years and older tested positive by qPCR. In the PBO-LLIN group, 12% (6/50) aged 0–4 years, 33.5% (56/167) aged 5–14 years and 29.4% (87/296) aged 15 years and older tested positive by qPCR.

### Factors associated with malaria infection prevalence

Among the variables evaluated at the individual level by logistic regression (Table 6), sex, sleeping under a net the previous night, and the age group 5–14 years significantly affected malaria prevalence. Males had a 1.5 times higher likelihood of malaria infection compared to females [aOR=1.517 (95% CI = 1.137–2.024), p = 0.005]. Those who slept under a net the previous night were 45.2% less likely to contract malaria compared to those who did not [aOR=0.548 (95% CI = 0.378–0.794), p = 0.001]. Children aged 5–14 years had a 1.9 times higher chance of malaria infection compared to children aged 0–4 years [aOR=1.901 (95% CI = 1.053–3.432), p = 0.033]. Being an adult (≥15 years) had no significant effect on malaria prevalence (p = 0.074). Intervention type had no significant effect on malaria prevalence, despite individuals in the PBO-LLIN group showing a 22.5% reduction in malaria prevalence [aOR=0.775 (95% CI = 0.582–1.032), p = 0.081].

**Table 6:** Factors associated with malaria infection.

Parameter	Details	Frequency (%), n = 963	Coefficient	aOR (95% CI)	*p*-value
Sex	Male	410 (42.6)	0.417	1.517 (1.137-2.024)	0.005
	Female	553 (57.4)	0	1	
Slept previous night	Yes	753 (78.2)	−0.601	0.548 (0.378-0.794)	0.001
	No	210 (21.8)	0	1	
Intervention	PBO-LLIN	513 (53.3)	−0.255	0.775 (0.582-1.032)	0.081
	Pyrethroid-LLIN	450 (46.7)	0	1	
Age group					0.103
	5-14 years	302 (31.4)	0.642	1.901(1.053-3.432)	0.033
	≥15 years	594 (61.7)	0.517	1.677 (0.950-2.960)	0.074

Dependent variable: malaria prevalence

Variables: Participant sex, sleeping under the net the previous night, Intervention (type of net), age group.

As a potential factor for outdoor malaria transmission, 97.2% (176/181) vs. 93% (185/199) households in Pyrethroid-LLINs and PBO-LLINs groups respectively reported night-time outdoor activities. The activities included cooking outside, early morning/late night market visits, farming, funeral activity, overnight fishing, security, taxi driving (boda boda), bar-attendance, roadside meetings, children playing outside among others.

## Discussion

The current study findings suggest that communities are more likely to own and use PBO-LLINs, which is critical for maintaining high coverage and effectiveness in malaria control especially in areas experiencing high pyrethroid resistance. Malaria prevalence was lower in the PBO-LLIN group, indicating its potential to reduce malaria transmission. According to a review conducted by Gleave and others [13], PBO nets have shown better efficacy against malaria when compared to standard LLINs. However, attainment of malaria control goal by this novel net depends on community ownership and usage. We demonstrate that bedbugs affect ownership and usage. Lack of proper beds also undermine net usage. Altogether, these factors negatively impact overall malaria control plan.

Attrition rates after one year were higher for Pyrethroid-LLINs compared to PBO-LLINs. In agreement that nets can be lost over time, a study in Ghana [33] reported 14.3% attrition of pyrethroid-LLINs within one year, while in Tanzania, 90% of Olyset Plus nets were lost due to wear and tear within three years [34]. In northwestern Tanzania, a study [34] evaluating net attrition observed the presence of new non-study nets during follow-up one year after net distribution. The primary reason for net loss in the study was wear and tear, which may have motivated households to replace study nets with new non-study nets. In contrast, the current study did not observe the presence of new non-study nets in the households. In another study [35] conducted in the malaria endemic regions of Kenya, at least 40% of nets were lost due to fabric decay. However, in the current study, the proportion of households reporting net loss due to wear and tear was relatively small. Since the study area is malaria-endemic, residents may have been more motivated to maintain and retain their nets due to heightened awareness of malaria risk and reinforced messaging about the superior effectiveness of PBO nets during distribution. This underscores the need for NMCP to prioritize net retention education and regular ownership checks for future PBO net distributions.

Fabric texture emerged as a significant factor in net preference in this study, with participants favouring softer fabrics over rougher textures, consistent with findings from Ghana [36], Cambodia [37], and India and Nepal [38]. However, this was not a predictor for net ownership. Future LLIN rollouts should consider fabric preferences and incorporate educational efforts to inform users about the benefits of different net types. Additionally, sleeping arrangements played a crucial role in net usage, with households possessing beds exhibiting significantly higher usage rates. Our findings corroborate with previous studies, such as Uganda [39], where net usage was consistently observed to be higher in households with proper beds compared to those using alternatives like papyrus mats or floor mattresses, which led to increased wear and tear due to frequent spreading and tucking of nets. Beds provided both functional benefits and aesthetic appeal when nets were hung. Similar observations were reported in Zambia’s Luapula and Northern provinces [40], where nets used over reed mats had significantly higher torn rates, with most holes concentrated on the bottom side, suggesting damage from contact with rough surfaces. A proper bed may also serve as a constant reminder to use a net, offering comfort while reducing abrasions that contribute to net deterioration. Given the variability in living conditions, community training on proper net care and usage remains essential, regardless of sleeping arrangements.

Bedbug infestation significantly influenced net ownership. Bedbug prevalence was slightly higher in Pyrethroid-LLIN households compared to PBO-LLIN households, suggesting that bedbugs might be resistant to the insecticides in the Pyrethroid-LLINs. However, PBO-LLINs were associated with a higher likelihood of ownership, indicating possible more effectiveness against bedbugs. In Ethiopia, presence of bedbugs also reduced ownership and usage of pyrethroid LLINs [41]. Similarly, in Eastern Rwanda, Kateera et al. [18] found that among reasons for the 13.8% LLINs non-use, bedbug infestation was reported. Bedbugs, being nocturnal, often use LLINs as hiding spots during the day, leading to the misconception that LLINs attract bedbugs. As determined by Hayes and Schal [42], bedbugs have shown high survival potential against pyrethroids. Bedbug bites can be irritating, and efforts to crush them causes blood stains on the nets thus requiring frequent washing of the nets [43], which may result in rapid insecticidal decay and damage due to constant tying and untying. The presence of bedbugs may also lead to net abandonment. As the NMCPs rollout PBO-LLINs widely in countries, it is important to incorporate bedbug management strategies so as to maximize on net usage.

In this study, individuals aged 15 years and older showed higher net usage compared to children under 5 years in the pyrethroid-LLIN group and children aged 5–14 years in the PBO-LLIN group. Males also used nets more frequently than females. This contrasts with Githinji et al. [44], which reported higher net usage among children under 5 years and females aged 15 years and older. In Southern Ethiopia, females were more likely to use their nets compared to males [45]. In our study, the perception that children under 5 years should sleep with their mothers, often leaving them without net protection when fathers sleep separately, highlights a cultural issue. Addressing these cultural norms through targeted educational and behavioral interventions is essential.

The PBO-LLIN group had a lower malaria prevalence than the pyrethroid-LLIN group, though the difference was not statistically significant. This is consistent with Staedke et al. [46], who reported prevalence of 11% in the PBO group compared to 13% in the pyrethroid-group within one year of net use in Uganda. However, in Northern Tanzania, Protopopoff et al. [47] demonstrated that PBO-LLINs significantly reduced malaria prevalence compared to pyrethroid-LLINs (69.3% vs. 80.9%, p-value: 0.0364) within 2.3 years. In Uganda, Sebuguzi et al. [48] determined that PBO-LLIN consistently posted lower malaria prevalence compared to pyrethroid-LLIN (22.7% vs 18.4%) in 25 months. From 2010–2014, a study in Nigeria [49] evaluating the cost effectiveness of PBO nets compared to the pyrethroid-LLINs, determined that the PBO nets reduced symptomatic malaria cases by 33.4% offering additional economic benefits. Consistent with the Kenya Malaria Indicator Survey 2021 [2], children aged 5–14 years had the highest malaria rates and used nets the least in PBO-LLIN group. Overall, LLIN use was associated with reduced malaria infections underscoring their importance in malaria prevention. The lower malaria rates, coupled with lower attrition rates, suggest potential long-term malaria control and highlight the need for improved net compliance strategies.

The current study addresses a critical gap in understanding community LLIN uptake and its effect on malaria transmission in an endemic setting. However, the study experienced some limitations as follows: (i) The findings may be influenced by confounders, such as the use of mosquito coils, burning herbs, or other local intervention practices that act as surrogates for LLINs. Future research evaluating PBO and other next-generation nets should account for these confounders to minimize bias. (ii) Data on the durability of the LLINs was not collected, which is crucial for future studies assessing the uptake and effectiveness of LLINs. Examining durability will provide a clearer understanding of long-term impact on malaria transmission. (iii) The reliance on self-reported data for net usage may be subject to recall or social desirability biases. To improve data reliability, future studies should explore methods for validating self-reported responses.

## Conclusion

This study demonstrates that PBO-LLINs had significantly higher ownership and usage rates compared to pyrethroid-LLINs one-year post-distribution. These findings suggest that PBO-LLINs may offer better community acceptance and retention, key factors in achieving Kenya’s malaria control goals of 100% net coverage and at least 80% usage. Additionally, the lower malaria prevalence observed among households using PBO-LLINs suggests possible efficacy, particularly in areas with pyrethroid-resistant mosquitoes. The study also highlights the role of household factors, such as bed ownership and bedbug infestations in influencing net ownership and usage. These findings underscore the need for ministries of health to enhance community education on proper net use, address barriers such as bed ownership, and integrate bedbug management strategies. Regular surveillance and targeted interventions are crucial to maximize the effectiveness of PBO-LLINs and achieve sustainable malaria control in endemic regions.

## Supporting information

S1 Checklist The ethical, cultural, and scientific considerations specific to inclusivity in global research. (DOCX)
